# Modified Newman and Friedman Extraoral Radiographic Technique

**Published:** 2012-06-01

**Authors:** Eshagali Saberi, Ladan Hafezi, Narges Farhadmolashahi, Manoochehr Mokhtari

**Affiliations:** 1. Department of Endodontics, Dental School, Zahedan University of Medical Sciences, Zahedan, Iran; 2. Department of Endodontics, Dental School, Azad University of Medical Sciences, Tehran, Iran; 3. Dentist, Zahedan, Iran

**Keywords:** Dental Radiography, Diagnostic Imaging, Diagnostic Techniques, Endodontics, Oral Radiology

## Abstract

**Introduction:**

Good radiographs are required for endodontic therapy and because some patient’s are intolerant to intraoral films and/or sensors, this can cause complications in endodontic treatment. Extraoral film placement can be used to obtain clinically diagnostic and working radiographs.

**Materials and Methods:**

The no. 2 receptor was placed against the model’s cheek and centered in the molar-premolar area. The central beam was directed toward this area from the opposite side. The vertical and horizontal angles that achieved the most accurate radiograph were calculated by trial and error.

**Results:**

The best method equated with the patient sitting upright and the Frankfort plane being horizontal to the floor and when the head was tilted 10 degrees toward the side being examined. For the upper posterior teeth the center of the image receptor was placed on the intersection of the ala-tragus and a parasagittal line while the upper border of receptor was parallel to the canthomeatal line; the cone was positioned a negative 25 degrees from the horizontal plane. The central beam was directed from midway between maxillary and mandibular premolars and molars of the opposite side. For the lower posterior teeth, the receptor was placed against the cheek on the side of interest and its lower border was parallel and 2 cm above the inferior border of the mandible. The cone was angled -20 degrees from the horizontal plane while the central beam was directed towards the mandibular molar-premolar region 1 cm below the lower border of the mandibular of the contralateral premolar/molar region.

**Conclusion:**

Using this novel technique, high quality images can be acquired for patients who cannot tolerate intraoral radiographs.

## Introduction

Radiographs are essential in endodontics they are a second set of “eyes” for the dentist. This is particularly true in endodontics, in which so many diagnostic and treatment decisions are based on radio-graphic findings [1].

The clinician has a variety of aids to facilitate a diagnostic radiograph. Most of these aids rely on conventional intraoral radiography. Some patients are unable to tolerate the conventional intraoral technique [2][3]. This group has increased in size with the advent of digital radiography. Extraoral film placement may be utilized while performing endodontic therapy for these patients [3][4]. This technique is an effective approach which can be used in the treatment of a wide spectrum of patients such as those with a developmentally disabled, trauma and trismus, exaggerated gag reflex, those of a young age, anatomical difficulties like large tongue, shallow palate, and restricted mouth opening as well as neurological difficulties [5][6][7]. For diagnostic purposes it can even be used on patients with severe dental phobia.

Newman and Friedman have introduced an extraoral radiographic technique for maxillary and mandibular teeth [3]. They reached a number of conclusions outlined below:

### Maxilla:

1) The patient should be sitting upright.

2) His/her mouth should be open as wide as possible. This allows the x-ray beam to pass to the sensor unobstructed from the opposite side of the mouth. Consequently, superimposition of the contralateral tissues on the image is avoided.

3) The sensor should be placed on the external surface of the cheek, directly buccal to tooth. A cotton roll is placed between the sensor and the cheek to parallel the sensor with the buccal surface of the tooth.

4) The x-ray cone should be angled approximately -55 degrees from the horizontal. Additionally, the x-ray cone must be aligned perpendicular to the sensor to provide an accurate image.

5) Increasing the exposure time may be necessary when conventional radiographs are used. Digital radiography may not require an increase in exposure time because the image can be adjusted digitally within radiographic software programs.

### Mandible:

1) The patient should be sitting upright.

2) The patient’s chin should be raised, which allows the x-ray beam to pass to the sensor unobstructed, thus avoiding superimposition of the contralateral tissues on the image.

3) The sensor should be placed on the external surface of the cheek, directly buccal to tooth. A cotton roll is placed between the sensor and the cheek to parallel the sensor with the buccal surface of the tooth.

4) The x-ray cone should be angled approximately 35 degrees from the horizontal. Additionally, the x-ray cone must be aligned perpendicular to the sensor to provide an accurate image.

5) Increasing the exposure time may be necessary.

Chen et al. introduced a special device for the adjustment of the x-ray with film/sensor and reported that the device can successfully be used in the extraoral technique. The most important points to consider in our study were the vertical and horizontal angles, which were different to Newman and Friedman study [8]. It is evident that their technique was introduced with vertical angles without any reference to anatomic landmarks for the points of entry of central rays and the exact location of the film or sensor, contrary to what is customary in oral radiology instructional procedures.

There are only a few studies that document the use of this technique. The aim of this study was to determine the exact points of entry for the x-rays and the location of the film or sensor based on anatomic landmarks for maxillary and mandibular molars and premolars.

## Materials and Methods

Initially, the exact location of receptor was determined based on the recommendations by Newman and Friedman on a head phantom. Then the relationship of this point with the anatomic landmarks (cranial planes and radiographic lines) was evaluated.

Subsequently, the x-ray entry points were determined based on the recommendations made by the two researchers on the head phantom and their relationship with anatomic landmarks was evaluated. Efforts were made to use different vertical and horizontal angles pre-determined on the x-ray tube to produce a radiograph with maximum image quality. Then the x-ray entry points were recorded with the new angles. Throughout the procedures, the head phantom was rotated approximately 10 degrees toward the radiographed side; similar to lateral oblique techniques.

In order to determine an appropriate exposure time, the exposure time recommended for extraoral techniques were initially used. This was reduced gradually until a high-quality radiograph was obtained. Finally, all the data collected for each arch was separately recorded and analyzed for maxillary and mandibular molars and premolars.

## Results

The trial and error method obtained these recommendations for taking high-quality extraoral radiographs:

### Maxillary premolars

**Figure 1:**
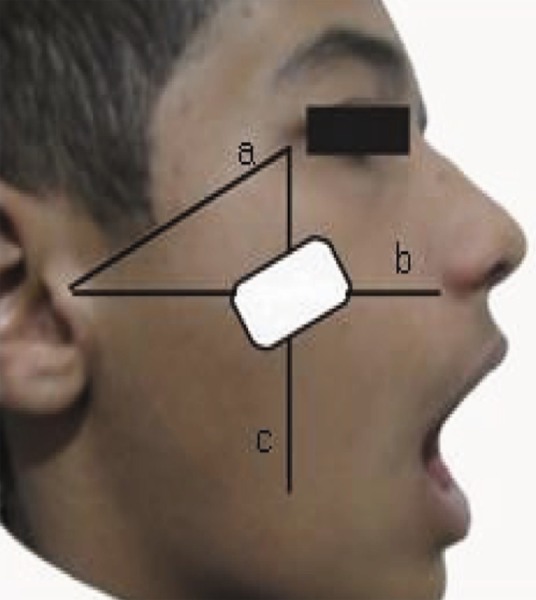
Location of the sensor for maxillary premolar and anatomical landmarks shown on patient’s profile (a. canthomeatal line; b. ala-tragus; c. parasagittal line)

**1) Patient position and image receptor:** the patient was sitting upright while the Frankfort plane [this plan extends from the upper border of the external auditory canal (anteriorly) to the upper border of the lower orbital rim [5]] was horizontal with the floor and mouth was wide open and the head was tilted approximately 10 degrees toward the side in question. The center of the image receptor was on the intersection of the ala-tragus and a parasagittal line (which begins from the outer canthus) and the upper border of the receptor parallel was to the canthomeatal line [this line joins the central point of the external auditory canal to the outer canthus of the eye [5] (Figure 1).

**Figure 2:**
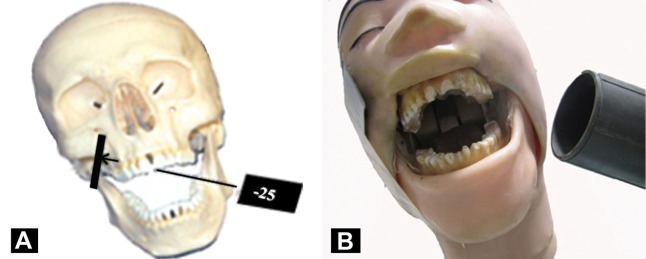
A) Angulation of the x-ray cone for the maxillary posterior: the cone is positioned a negative 25° from the horizontal plane. Place a no. 2 receptor against the phantom’s cheek; B) The head is tilted 10° toward the side being examined

**2) Position of the central x-ray beam:** the x-ray cone was angled approximately -25 degrees from the horizontal plane while the central beam was directed midway between maxillary and mandibular premolars on the opposite side to the center of the image receptor (Figure 2). An intraoral x-ray machine was used to take the radiograph set at 66KVP, 8 MA, 0.7 seconds.

### Maxillary molars

**1) Patient position and image receptor:** the position was like the maxillary premolars. The center of the image receptor was on the intersection of the ala-tragus and parasagittal line (beginning 1 cm posterior to the outer canthus) and the upper border of the receptor was parallel to the canthomeatal line.

**2) Position of the central x-ray beam:** the x-ray cone was angled approximately -25 degrees from the horizontal plane while the central ray was directed midway between maxillary and mandibular molars on the opposite side to the center of the image receptor (Figure 2).

### Mandibular premolars and molars

**1) Patient position and image receptor:** the same position with maxillary teeth is obtained. The receptor is placed against the patient's cheek on the side of interest and its lower border was parallel and at least 2 cm above the inferior border of the mandible (the lower border of the receptor was approximately at the CEJ of the tooth to be radiographed.)

**2) Position of the central x-ray beam:** the x-ray cone is angled approximately -20 degrees from the horizontal plane while the central beam was directed from 1 cm below the lower border of the mandible at the premolar/molar area contralateral to the center of the receptor.

## Discussion

Although extraoral radiography should not and will not replace conventional intraoral radiography, it is a useful supplement for clinical practice. It is an efficient technique for achieving diagnostic films in particular patients. The technique is a sample method that allows the clinician to capture an appropriate image for patients who are unable to tolerate the placement of intraoral films or sensors. This technique may be utilized with the rubber dam in place, making it applicable for all phases of endodontic therapy [3][6]. The advantage of this technique is the increased patient compliance providing images with adequate details and diagnostic quality. However, the procedure is technique sensitive, has slightly lower image resolution, and unable to obtain radiographs of anterior teeth [7].

In 1974, Fisher proposed an extraoral radiographic technique for obtaining images of third molars using occlusal film, however, the requisite high KVP (as high as 90 KVP) had limitations in its daily clinical application [6]. We found that, using a digital imaging system at 66 KVP was sufficient to produce diagnostic quality image comparable with the conventional intraoral periapical radiographs.

Obtaining an appropriate extraoral radiograph during endodontic treatment is difficult [3] because of the long distance between the x-ray source and the receptor and therefore we occasionally have to expose the patient several times to x-rays which leads to ethical considerations and problems. The most important problem with the technique–despite its advantages–is that x-ray entry points and exact location of receptor based on anatomic landmarks have not been separately specified for each tooth. Although the device introduced by Chen et al. solves the problem to a great extent [8], we carried out this study to collect detailed information regarding the technique for dental students and practitioners when the device is not available.

**Figure 3:**
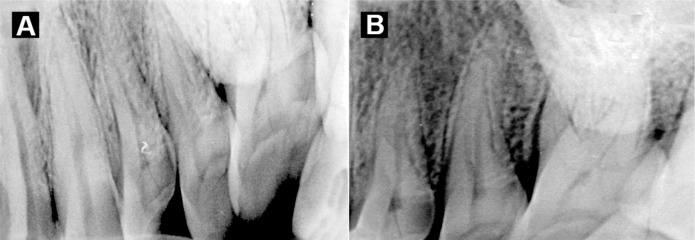
Resultant images of maxillary posterior region

This study was carried out on a head phantom in an effort to exactly determine the image receptor and patient placement, central beam direction and exposure times, separately for maxillary and mandibular molars and premolars so that dental students and clinicians can obtain high quality radiographs at the shortest possible time (Figure 3). The main anatomic landmark used in patient positioning during extraoral radiography is the canthomeatal line which forms approximately a 10 degree angle with the Frankfort plane. When digital techniques are used exposure times are lowered to the minimum [5].

Although x-ray tube angulations yielded by the present study are different from the ones reported by Newman and Friedman [3], they are close to the ones reported by Chen et al., which might be attributed to the rotation of the phantom head toward the side being radiographed in the present study (similar to lateral oblique technique), and possibly to racial differences in facial height [8].

Radiological techniques play an important role in measurements of anatomic landmarks [9], degree of canal curvature [10], detection of voids [11], procedural accidents and errors [12], determining working length [13], as well as treatment outcome interpretation [14][15]. With recent advances in dental radiography, various techniques like panoramic radiography are accessible to manage difficult patients; however, this novel technique can be recommended where panoramic radiographs are not available.

## Conclusion

Extraoral radiography technique can be a very useful diagnostic procedure in patients which cannot have intraoral radiographs. We recommend further standardization of this technique for superior image quality.
